# Accelerated Stochastic Variance Reduction Gradient Algorithms for Robust Subspace Clustering

**DOI:** 10.3390/s24113659

**Published:** 2024-06-05

**Authors:** Hongying Liu, Linlin Yang, Longge Zhang, Fanhua Shang, Yuanyuan Liu, Lijun Wang

**Affiliations:** 1 Medical College, Tianjin University, Tianjin 300072, China; hyliu2009@tju.edu.cn; 2 Peng Cheng Laboratory, Shenzhen 518000, China; 3 Key Laboratory of Intelligent Perception and Image Understanding of Ministry of Education, School of Artificial Intelligence, Xidian University, Xi’an 710071, China; 20171110340@stu.xidian.edu.cn (L.Y.); yyliu@xidian.edu.cn (Y.L.); 4 Department of Mathematics and Physics, North China Electric Power University, Baoding 071003, China; 5 College of Intelligence and Computing, Tianjin University, Tianjin 300350, China; fhshang@tju.edu.cn; 6 Hangzhou Institute of Technology, Xidian University, Hangzhou 311231, China

**Keywords:** sparse subspace clustering, face clustering, stochastic optimization, variance reduction

## Abstract

Robust face clustering enjoys a wide range of applications for gate passes, surveillance systems and security analysis in embedded sensors. Nevertheless, existing algorithms have limitations in finding accurate clusters when data contain noise (e.g., occluded face clustering and recognition). It is known that in subspace clustering, the $\ell_{1}$- and $\ell_{2}$-norm regularizers can improve subspace preservation and connectivity, respectively, and the elastic net regularizer (i.e., the mixture of the $\ell_{1}$- and $\ell_{2}$-norms) provides a balance between the two properties. However, existing deterministic methods have high per iteration computational complexities, making them inapplicable to large-scale problems. To address this issue, this paper proposes the first accelerated stochastic variance reduction gradient (RASVRG) algorithm for robust subspace clustering. We also introduce a new momentum acceleration technique for the RASVRG algorithm. As a result of the involvement of this momentum, the RASVRG algorithm achieves both the best oracle complexity and the fastest convergence rate, and it reaches higher efficiency in practice for both strongly convex and not strongly convex models. Various experimental results show that the RASVRG algorithm outperformed existing state-of-the-art methods with elastic net and $\ell_{1}$-norm regularizers in terms of accuracy in most cases. As demonstrated on real-world face datasets with different manually added levels of pixel corruption and occlusion situations, the RASVRG algorithm achieved much better performance in terms of accuracy and robustness.

## 1. Introduction

Subspace clustering aims to find groups of similar objects or clusters, which usually exist in low-dimensional subspaces. With the devolvement of artificial intelligence and the popularity of computer vision applications such as face recognition and clustering [1,2], motion segmentation [3] and document analysis [4], subspace clustering has attracted more attention in recent years, especially mask-occluded face recognition due to COVID-19 in embedded sensors. Samples of different classes can be approximated well by data from a union of low-dimensional subspaces. In practice, we perform the task of dividing the data points based on their similarity into various subspaces. Subspace clustering is one subcategory of clustering which gathers data into different groups until each group consists of data points from the same subspace only. For subspace clustering, there are series of related methods being developed, such as statistical, iterative and algebraic methods, spectral clustering and deep learning algorithms [5,6,7,8,9,10].

Compared with other techniques, the methods based on spectral clustering have become increasing popular because of convenient implementation, complete theoretical support and reliable accuracy [11]. The key of these methods is to adopt an $\ell_{1},\ell_{2}$-norm or elastic net (i.e., a mixture of $\ell_{1}$- and $\ell_{2}$-norms) regularizer to solve an optimization problem for obtaining an affinity matrix and making further use of spectral clustering with the matrix. Each point from the union of subspaces can be represented as a linear combination of other data points in the subspace [12], which is called the self-expressiveness property. It can be formulated as follows:

(1) $$x_{j}=Xc_{j}\;\mathrm{and}\;c_{jj}=0.$$

In fact, Equation (1) is equivalent to the following form:

(2) X=XCanddiag(C)=0

where $X\;=\;\left[x_{1},x_{2},\ldots ,x_{N}\right]\;\in \;R^{D\times N}$ is the data matrix, whose *j*th column corresponds to the sparse representation of $x_{j}$, $C\;=\;\left[c_{1},c_{2},\ldots ,c_{N}\right]\;\in \;R^{N\times N}$ is the coefficient matrix, whose *j*th column corresponds to $c_{j}$, $c_{jj}$ is the *j*th element of $c_{j}$ and $\mathrm{diag}\left(C\right)\;\in \;R^{N}$ is the vector of the diagonal elements of *C*. Although multiple sets of solutions for *C* may be found rather than a unique solution, such a solution under the condition that if $c_{ij}\;\neq \;0$, then $x_{i}$ belongs to the same subspace as $x_{j}$ still exists. Due to the reservation of subspace clustering, these solutions are called subspace-preserving. If a subspace-preserving *C* exists, and the connection between a pair of points $x_{i}$ and $x_{j}$ can be established in an affinity matrix *W* (i.e., $w_{ij}\;=\;\left|c_{ij}\right|\;+\;\left|c_{ji}\right|$), then one can cluster the data points by applying spectral clustering [13] to the affinity matrix.

In order to obtain the subspace-preserving matrix *C*, one effective method is to regularize *C* and solve the following minimization problem:

(3) $$C^{*}=\underset{C}{\arg\min}\;{∥C∥}_{1},\;s.t.,\;X=XC,\;\;\mathrm{diag}\left(C\right)=0$$

where ${∥\;\cdot \;∥}_{1}$ is the $\ell_{1}$-norm (i.e., ${∥C∥}_{1}\;=\;\sum_{i=1}^{n}\;\sum_{j=1}^{n}\;\left|c_{ij}\right|$). Here, the $\ell_{1}$-norm can be replaced by the $\ell_{2}$-norm (i.e., ${∥C∥}_{2}\;=\;\sqrt{\sum_{i=1}^{n}\;\sum_{j=1}^{n}\;c_{ij}^{2}}$).

Due to the choice of regularizers for the coefficient matrix *C*, there are different subspace clustering algorithms. For instance, the sparse subspace clustering (SSC) method [12] applies the $\ell_{1}$-norm to find the coefficient matrix *C*. Previous work has indicated that SSC can provide a subspace-preserving solution under certain circumstances, where the subspaces are independent [14] or the data in different subspaces meet some separation conditions, and the data in the same subspaces distribute well [14,15]. Similar conclusions are also obtained when data are corrupted by noise [16] or outliers [17]. Least squares regression [18] uses the $\ell_{2}$-norm regularizer on the matrix *C*. Low-rank representation [19] applies the nuclear norm regularizer to *C* to retain its low-rank property. Moreover, the authors of [17,20,21] utilized the elastic net regularizer to induce a sparse matrix *C*.

In recent years, many subspace clustering methods have greatly promoted the development of SSC algorithms. However, the performance of these methods is mostly evaluated in a case where the datasets are clean, ignoring the existence of potential noise in reality. On the other hand, many algorithms require additional procedures to evaluate noises and remove them. For instance, the authors of [22] required principal components analysis (PCA) to be performed on the data for dimensionality reduction and noise reduction, and the authors of [17] modeled and removed the outliers for further clustering. These methods have a strong dependence on the cleanliness of the data. Therefore, in consideration of the above reasons, the actual performance of existing methods on real-world datasets is not always satisfied.

In this paper, we propose a robust accelerated stochastic variance reduction gradient (RASVRG) method and present its efficient implementation for self-expressiveness-based subspace clustering problems. Our algorithms can be directly applied to SSC problems with $\ell_{1}$-norm and elastic-net regularizers on datasets that may be corrupted by potential noise and achieve superior clustering accuracy and efficiency compared with existing popular algorithms, demonstrating the excellent performance and strong robustness of the RASVRG.

The key accelerated technique in the RASVRG is the snapshot momentum proposed in our previous work [23,24]. We introduce the momentum acceleration technique into the proximal stochastic variance reduction gradient (Prox-SVRG) method [25]. The proposed RASVRG algorithms require tracking only one variable vector in the inner loop, which means that its computational time and memory overhead are exactly the same as those of the SVRG [26] and Prox-SVRG [25]. Thus, our RASVRG algorithms have much lower per iteration complexity than other accelerated methods (e.g., Katyusha [27]), which means that the RASVRG is more suitable for large-scale SSC problems [28], especially large-scale robust face clustering problems. To the best of our knowledge, this work is the first one to propose faster stochastic optimization algorithms instead of deterministic methods to solve various SSC problems, including robust face clustering.

We summarize the major contributions of this paper as follows:

1. **Faster convergence rates:** Our RASVRG obtains the oracle complexity $O(\left(D\;+\;\sqrt{D\kappa }\right)\log\left(1/\epsilon \right))$ for strongly convex (SC) subspace clustering problems (e.g., the elastic net regularized face clustering problem), which is the best oracle gradient complexity, as pointed out in [29], where κ is the condition number of the objective function. For subspace clustering problems which are not strongly convex (non-SC) (e.g., the $\ell_{1}$-norm regularized problem), the RASVRG achieves an optimal convergence rate $O(1/S^{2})$, where *S* is the number of epochs; that is, the RASVRG is much faster than existing stochastic and deterministic algorithms, such as the Prox-SVRG [25].
2. **Better accuracy:** Both in theory and in practice, our algorithms can generally yield better performance than existing state-of-the-art methods for solving problems with the $\ell_{1}$-norm or elastic net regularizer, while most existing methods are greatly influenced by the choice of the regularizer.
3. **More robust:** Our RASVRG obtains much better performance compared with other algorithms on real-world datasets with different manually added levels of random pixel corruption or unrelated block occlusion for simulating potential real-world noise, while existing methods may be seriously deteriorated by such strong noise.
4. **Extension to more applications:** Our RASVRG requires tracking only one variable vector in the inner loop, which means its computational cost and memory overhead are exactly the same as the SVRG [26] and Prox-SVRG algorithms. This feature allows the RASVRG to be extended to other real-world clustering applications and more settings such as the sparse and asynchronous setting, which can significantly accelerate the speed of the RASVRG.

The rest of this paper is organized as follows. In Section 2, we discuss some related works concerning sparse subspace clustering and stochastic optimization methods. Section 3 proposes two efficient RASVRG algorithms for solving both strongly convex and non-strongly convex models and analyzes their convergence properties for $\ell_{1}$-norm and elastic net regularized SSC problems. In Section 4, we exhibit the practical performance of the RASVRG for subspace clustering tasks on synthetic and real-world face datasets. Section 5 concludes this paper and discusses future work.

## 2. Related Works

In this section, we briefly overview some related works concerning sparse subspace clustering and stochastic optimization.

### 2.1. Sparse Subspace Clustering

In this part, we briefly overview sparse subspace clustering (SSC) [22]. Let $X=\left[x_{1},\cdots ,x_{N}\right]\in R^{D\times N}$ be a union of *N* signals ${\left\{x_{i}\in R^{D}\right\}}_{i=1}^{N}$ from a union of *K* linear subspaces $S_{1}\cup S_{2}\cup \cdots \cup S_{n}$ and ${\left\{d_{k}\right\}}_{k=1}^{K}$∈$R^{D}$. In addition, $X_{k}\in R^{D\times N_{k}}$ is a submatrix of *X* and $N_{k}$ points in the subspace $S_{k}$ which satisfies $\sum_{k=1}^{K}\;N_{k}\;=\;N$.

Each point from a random subspace $S_{k}$ can be represented by a linear combination of at most $\left(N\;-\;N_{k}\right)$ other points from other subspaces. Therefore, we can find it by solving the following optimization problem:

(4) $$\min∥c_{j}{∥}_{0},\;s.t.,\;x_{j}=Xc_{j},c_{jj}=0$$

where $c_{j}\;=\;{\left[c_{j1},c_{j2},\cdots ,c_{jN}\right]}^{T}\;\in \;R^{N}$ are the coefficient vectors, and $∥c_{j}{∥}_{0}$ counts the number of nonzero entries in vector $c_{j}$. Since this is an NP-hard problem, the authors of [12] relaxed this problem and solved the following problem:

(5) $$\min∥c_{j}{∥}_{1},\;s.t.,\;x_{j}=Xc_{j},c_{jj}=0$$

where ${∥c_{j}∥}_{1}\;=\;\sum_{i=1}^{N}\left|c_{ji}\right|$ is the $\ell_{1}$-norm of $c_{j}\;\in \;R^{N}$. For all the data points i=1,⋯,N, the optimization problem (Equation (5)) can be expressed in matrix form:

(6) $$\min{∥C∥}_{1},\;s.t.,\;X=XC,\;\mathrm{diag}\;\left(C\right)=0$$

where $C\;=\;\left[c_{1},c_{2},\ldots ,c_{N}\right]\;\in \;R^{N\times N}$ is the coefficient matrix and each column $c_{i}$ is the sparse representation vector of $x_{i}$.

Equations (4) and (5) have attracted much attention in the fields of compressed sensing [30,31], subspace clustering [5] and face recognition [32]. In fact, the two solutions are the same under certain conditions. However, the results of compressed sensing may not be suitable for the subspace clustering problem, since the solution for *C* is not necessarily unique as the columns of *X* lie in a union of subspaces. When the matrix *C* is obtained, spectral clustering [33] is adopted in the affinity matrix $W\;=\;|C|+{\left|C\right|}^{T}$ for clustering.

Furthermore, we also consider the case where the data points from a union of linear subspaces contain a certain amount of noise. More specifically, the *j*th data point contaminated with noise $\zeta_{j}$ is represented by ${\bar{x}}_{j}\;=\;x_{j}\;+\;\zeta_{j}$, where $\zeta_{j}$ satisfies the condition of ${∥\zeta_{j}∥}_{2}\;\leq \;\epsilon$ and ${∥\;\cdot \;∥}_{2}$ is the Euclidean norm. We can find the sparsest solution of the following problem to obtain the sparse representation of ${\bar{x}}_{j}$ with a given error tolerance ϵ:

(7) $$\min{∥c_{j}∥}_{1}\;\;s.t.,\;\;{∥Xc_{j}-{\bar{x}}_{j}∥}_{2}\leq \epsilon ,\;c_{jj}=0.$$

However, we cannot acquire the scale of the noise $\zeta_{j}$ in most instances. Under this circumstance, the Lasso optimization algorithm [34] can be applied to obtain the sparse representation in the following form:

(8) $$\min{∥c_{j}∥}_{1}+\gamma {∥Xc_{j}-{\bar{x}}_{j}∥}_{2}^{2},\;c_{jj}=0$$

where γ≥0 is a constant parameter.

In addition, potential connectivity issues in the representation graph may exist [35] (i.e., over-segmentation problems). This phenomenon is caused by sparsity of the representation matrix *C* computed from Equation (8). In order to promote connections between data points, the authors of [17,21] used the elastic net regularizer to solve the sparse coefficient matrix *C* as follows:

(9) $$\min_{c_{j}}\;\lambda {∥c_{j}∥}_{1}+\frac{1-\lambda }{2}{∥c_{j}∥}_{2}^{2}+\frac{\gamma }{2}{∥Xc_{j}-{\bar{x}}_{j}∥}_{2}^{2}$$

where λ∈[0,1] determines the trade-off between sparseness (from the $\ell_{1}$-norm regularizer) and connectivity (from the $\ell_{2}$-norm regularizer). In particular, when λ is extremely close to one, the performance of the elastic net approaches is much better than that of the method based on the $\ell_{1}$-norm. The purpose of the $\ell_{2}$-norm regularizer is to enhance the connectivity between data points; that is, in the case of a relatively small λ, there exist more nonzero elements in the matrix *C*.

### 2.2. Stochastic Methods

All the algorithms mentioned above for SSC are deterministic methods, and their per iteration complexity is O(ND), which is expensive for extremely large *N* values. Recently, stochastic gradient descent (SGD) has been successfully applied to many large-scale machine learning problems due to its significantly lower per iteration complexity O(D). SGD only requires one (or a small batch of) component function(s) per iteration to form an estimator of the full gradient. However, the variance of the stochastic gradient estimator may be large [26], which leads to slow convergence and poor performance.

More recently, many researchers have proposed accelerated stochastic variance reduced methods such as Acc-Prox-SVRG [36], APCG [37], Catalyst [38], SPDC [39], point-SAGA [40], Katyusha [27] and MiG [23]. For strongly convex problems, both Acc-Prox-SVRG [36] and Catalyst [38] make good use of Nesterov’s momentum in [41] and attain the corresponding oracle gradient complexities $O(\left(D\;+\;b\sqrt{\kappa }\right)\log\left(1/\epsilon \right))$ with a sufficiently large mini-batch size *b* and $O(\left(D\;+\;\sqrt{\kappa D}\right)\log\left(\kappa \right)\log\left(1/\epsilon \right))$, respectively, where $\kappa =\frac{L}{\sigma }$ denotes the condition number of an *L*-smooth and σ strongly convex function. In particular, APCG, SPDC, point-SAGA and Katyusha essentially achieve the best-known oracle complexity $O(\left(D\;+\;\sqrt{\kappa D}\right)\log\left(1/\epsilon \right))$. For non-strongly convex problems, Katyusha also attains the best-known convergence rate $O(1/S^{2})$. However, existing accelerated stochastic variance reduction methods generally have more complex update rules and higher computational costs [42]. Therefore, this paper will propose a faster stochastic variance reduced gradient method for sparse subspace clustering.

## 3. Accelerated Stochastic Variance Reduced Gradient Algorithms for Sparse Subspace Clustering

In this section, we propose a new robust accelerated stochastic variance reduced gradient (RASVRG) method for solving sparse representation problems such as SSC. For elastic net regularized problems, we present a strongly convex (SC) version of the RASVRG (RASVRG$\;{\;}^{\mathrm{SC}}$) which has the best-known linear convergence rate. Moreover, we also provide a non-strongly convex (NSC) version (RASVRG$\;{\;}^{\mathrm{NSC}}$) which attains the fastest convergence rate $O(1/S^{2})$.

Compared with stochastic variance reduced gradient methods (e.g., SVRG [26] or SAGA [43]), most existing accelerated methods have improved convergence rates while having complex coupling structures, which leads to slow convergence in practice [24]. Thus, we propose a robust accelerated stochastic variance reduced gradient (RASVRG) method for both strongly convex and non-strongly convex problems. This means that the RASVRG can solve the SSC problem [14] based on the $\ell_{1}$-norm regularizer and elastic net regularizer [21]. We focus on the following convex optimization problem with a finite-sum structure, which is a common problem in machine learning and statistics:

(10) $$\min_{\alpha \in R^{d}}\left\{F\left(\alpha \right)=f\left(\alpha \right)+g\left(\alpha \right)\right\}.$$

Here, the symbol α stands for the parameter, and *N* is the dimension of α. Meanwhile, $f\left(\alpha \right)=\frac{1}{D}\sum_{i=1}^{D}f_{i}\left(\alpha \right)$ is a finite average of *D* smooth convex functions $f_{i}\left(x\right)$, and g(x) is a relatively simple convex function. A convex function $f:R^{N}\to R$ is *L*-smooth if for all $\alpha ,\beta \in R^{N}$, we have

(11) $$f\left(\alpha \right)\leq f\left(\beta \right)+\langle \nabla \;f\left(\beta \right),\alpha -\beta \rangle +\frac{L}{2}{∥\alpha -\beta ∥}^{2}$$

and it is σ strongly convex if for all $\alpha ,\beta \in R^{d}$, we have

(12) $$f\left(\alpha \right)\geq f\left(\beta \right)+\langle G,\alpha -\beta \rangle +\frac{\sigma }{2}{∥\alpha -\beta ∥}^{2}$$

where G∈∂f(α) is a sub-gradient of *f* at α. We make the following two assumptions to categorize Equation (10):

**Assumption** **1** (Strongly convex)**.** *In Equation (10), each $f_{i}\left(\cdot \right)$ is L-smooth and convex, and g(·) is σ strongly convex.*

**Assumption** **2** (Non-strongly convex)**.** *In Equation (10), each $f_{i}\left(\cdot \right)$ is L-smooth and convex, and g(·) is convex.*

Next, we propose an efficient RASVRG method for both strongly convex (e.g., elastic net regularized SSC) and non-strongly convex (e.g., $\ell_{1}$-norm regularized SSC) problems.

### 3.1. RASVRG$\;{\;}^{\mathrm{SC}}$ for Elastic Net Regularized SSC

In this subsection, we consider the elastic net regularized SSC problem, which is strongly convex. Inspired by our previous work [23], we present the RASVRG$\;{\;}^{\mathrm{SC}}$, shown in Algorithm 1, as a solver together with an active set-based optimization framework [21] (i.e., Oracle Guided Elastic Net (ORGEN)):

(13) $$f\left(c;b,X\right)\;:=\;\lambda {∥c∥}_{1}\;+\;\frac{1\;-\;\lambda }{2}{∥c∥}_{2}^{2}\;+\;\frac{\gamma }{2}{∥b\;-\;Xc∥}_{2}^{2},\;\;c_{jj}\;=\;0$$

where $b\;\in \;R^{D}$, $X\;=\;\left[x_{1},\ldots ,x_{N}\right]\;\in \;R^{D\times N}$, γ≥0 and λ∈[0,1). In addition, b and ${\left\{x_{j}\right\}}_{j=1}^{N}$ are normalized to an $\ell_{2}$-norm unit. The goal of the elastic net model is to attain $c^{*}\left(b,X\right)$ as follows:

(14) $$c^{*}\left(b,X\right):=\underset{c}{\arg\min}\;f\left(c;b,X\right).$$

We apply our RASVRG$\;{\;}^{\mathrm{SC}}$ to compute $c^{*}\left(b,X\right)$. As we can see in step 5 of Algorithm 1, *y* is a convex combination of *c* and $\tilde{c}$ with the momentum parameter θ. In other words, our algorithm uses the momentum acceleration technique proposed in our previous work [23]. We just need to keep track of *y* in a single inner loop and have a fancy update for $\tilde{c}$ (see step 10), which is efficient but simple in implementation. Note that temporary variables $w_{1}$ and $w_{2}$ are also introduced into Algorithm 1, which will make our algorithm statement clearer. Of course, we can rewrite the algorithm and keep track of only one vector per iteration during implementation. Below, we give the convergence analysis of the RASVRG$\;{\;}^{\mathrm{SC}}$.
**Algorithm 1** RASVRG$\;{\;}^{\mathrm{SC}}$.**Input:** Initial vector $c_{0}$, train matrix $A\;\in \;R^{D\times (N-1)}$, $b\;\in \;R^{D}$, epoch length *m*, learning rate η and parameters θ and γ.**Initialize:** ${\tilde{c}}_{0}=c_{0}^{1}=c_{0}$, p=1+ησ.1:**for** s=1…S **do**2:   $\mu_{s}=\frac{\gamma }{D}A^{T}\left(A{\tilde{c}}_{s-1}-b\right)$;3:   **for** j=1…m **do**4:     Pick a row $i_{j}\in \left\{1\ldots D\right\}$ randomly from *A* and assign it to $a^{T}$;5:     $y_{j}=\theta c_{j-1}^{s}+\left(1-\theta \right){\tilde{c}}_{s-1}$;6:     $w_{1}\;=\;a\left(a^{T}y_{j}\;-\;b_{i_{j}}\right)\;-\;a\left(a^{T}{\tilde{c}}_{s-1}\;-\;b_{i_{j}}\right)\;+\;\mu_{s}\;+\;\frac{1-\lambda }{\gamma }c_{j-1}^{s}$;7:     $w_{2}=c_{j-1}^{s}-\eta \times w_{1}$;                         //*temp variable $w_{1},w_{2}$*8:     $c_{j}^{s}=\mathrm{sign}\left(w_{2}\right)\max\{|w_{2}\left|-\frac{\lambda }{\gamma }\eta ,0\right\}$;9:   **end for**10:   ${\tilde{c}}_{s}=\theta {\left(\sum_{j=0}^{m-1}p^{j}\right)}^{-1}\sum_{j=0}^{m-1}\omega^{j}c_{j+1}^{s}+\left(1-\theta \right){\tilde{c}}_{s-1}$;11:   $c_{0}^{s+1}=c_{m}^{s}$;12:**end for****Output:** ${\tilde{c}}_{s}$.

**Theorem** **1** (Strongly convex)**.** Let $c^{*}$ be the optimal solution for Equation (13). Suppose that Assumption 1 holds. Then, by choosing m=Θ(D), the RASVRG$\;{\;}^{\mathrm{SC}}$ achieves an ϵ-additive error with the following oracle complexity in expectation:

(15) $$\left\{\begin{array}{cc} O\left(\sqrt{\kappa D}\log\frac{F\left(c_{0}\right)-F\left(c^{*}\right)}{\epsilon }\right), & \;\mathrm{if}\;\frac{m}{\kappa }\leq \frac{3}{4},\\ O\left(D\log\frac{F\left(c_{0}\right)-F\left(c^{*}\right)}{\epsilon }\right), & \;\mathrm{if}\;\frac{m}{\kappa }>\frac{3}{4}. \end{array}\right.$$

The proof of this theorem is similar to that in our previous work [23], and thus it is omitted here. Similar to the analysis in our previous work [23], the overall oracle complexity of the RASVRG$\;{\;}^{\mathrm{SC}}$ is $O\left(\left(D\;+\;\sqrt{\kappa D}\right)\log\frac{1}{\epsilon }\right)$. This result indicates that under strongly convex conditions, the RASVRG$\;{\;}^{\mathrm{SC}}$ has the best-known oracle complexity for stochastic accelerated algorithms (e.g., APCG [37], SPDC [39] and Katyusha [27]). As analyzed in [23], the RASVRG$\;{\;}^{\mathrm{SC}}$ has only one variable *y*, while most existing accelerated methods, including Katyusha, require two additional variables. Therefore, the RASVRG$\;{\;}^{\mathrm{SC}}$ has a faster convergence speed than them in practice.

We applied the RASVRG$\;{\;}^{\mathrm{SC}}$ to the ORGEN framework, which is an efficient method that can handle large-scale datasets. We could find the optimal $c^{*}$ of each column of *X*. The basic idea of ORGEN is to solve a sequence of reduced-scale subproblems defined by an active set. Here, we introduce the ORGEN-RASVRG$\;{\;}^{\mathrm{SC}}$ algorithm, as shown in Algorithm 2.
**Algorithm 2** ORGEN-RASVRG$\;{\;}^{\mathrm{SC}}$.**Input:** $A\in R^{D\times (N-1)}$, $b\in R^{D}$, λ and γ.**Initialize:** The support set $T_{0}$ and set k←0.1:**while** $T_{k+1}⊈T_{k}$ **do**2:   Compute $c^{*}\left(b,A_{T_{k}}\right)$ by using Algorithm 1;3:   Compute $\delta \left(b,A\right):=\gamma \cdot \left(b-Ac^{*}\left(b,A\right)\right)$;4:   Update the active set: $T_{k+1}\leftarrow \left\{j:a_{j}\in \delta \left(b,A_{T_{k}}\right)\right\}$;5:   Set k←k+16:**end while****Output:** *c*: $c_{T_{k}}\;=\;c^{*}\;\left(b,A_{T_{k}}\right)$ and zeros otherwise.

By solving a series of reduced-scale problems in step 2 of Algorithm 2, we can address large-scale data efficiently. Next, we define the subspace clustering problem with the ORGEN-RASVRG$\;{\;}^{\mathrm{SC}}$. Let $X\;\in \;R^{D\times N}$ be a real value matrix whose columns are drawn from a union of *n* subspaces of $R^{D}$, where each $x_{i}$ is normalized to be an $\ell_{2}$-norm unit. Here, we use $A\;=\;X_{j}$ to denote the matrix *X* without the *j*th column. The goal of subspace clustering is to segment each column of *X* into their corresponding subspaces by finding a sparse representation of each point in terms of other points. The sparse subspace clustering procedure of the ORGEN-RASVRG$\;{\;}^{\mathrm{SC}}$ is shown in Algorithm 3.
**Algorithm 3** SSC by ORGEN-RASVRG$\;{\;}^{\mathrm{SC}}$.**Input:** $\mathrm{Data}X=\left[x_{1},\ldots ,x_{N}\right]$ and parameters $k_{\max}$ and ϵ.  $b\leftarrow x_{j}$, $A\leftarrow X_{j}$, and compute $c^{*}\left(b,A\right)$ by ORGEN-RASVRG$\;{\;}^{\mathrm{SC}}$;  Set $C^{*}=\left[c_{1}^{*},\cdots ,c_{N}^{*}\right]$ and $W=\left|C^{*}\right|+\left|C^{*⊤}\right|$;  Compute segmentation from *W* by spectral clustering;**Output:** Segmentation of data *X*.

The vector $c_{j}^{*}\;\in \;R^{N}$ (i.e., the *j*th column of $C^{*}\;\in \;R^{N\times N}$) is computed by the ORGEN-RASVRG$\;{\;}^{\mathrm{SC}}$. After $C^{*}$ was computed, and by using spectral clustering for the matrix $W=\left|C^{*}\right|+\left|C^{*⊤}\right|$, we then obtained the segmentation result of *X*.

### 3.2. RASVRG$\;{\;}^{\mathrm{NSC}}$ for $\ell_{1}$-Norm Regularized SSC

In this subsection, we consider Equation (13) with λ=1, also known as the Lasso problem, which is a non-strongly convex problem:

(16) $$f\left(c;b,X\right):={∥c∥}_{1}+\frac{\gamma }{2}{∥b-Xc∥}_{2}^{2}.$$

The RASVRG$\;{\;}^{\mathrm{NSC}}$ shown in Algorithm 4 can achieve a convergence rate of $O\left(1/S^{2}\right)$.
**Algorithm 4** RASVRG$\;{\;}^{\mathrm{NSC}}$.**Input:** $A\in R^{D\times (N-1)}$, $b\in R^{D}$, γ and epoch length *m*.**Initialize:** ${\tilde{c}}_{0}=c_{0}^{1}=c_{0}$;1:**for** s=1…S **do**2:   $\theta \;=\;\frac{2}{s+4}$, $\eta \;=\;\frac{1}{4L\theta }$, $\tilde{c}\;=\;{\tilde{c}}_{s-1}$, $\mu_{s}\;=\;\frac{\gamma }{D}A^{T}\left(A{\tilde{c}}_{s-1}\;\;-b\right)$;3:   **for** j=1…m **do**4:     Pick a row $i_{j}\in \left\{1\ldots D\right\}$ randomly from *A* and assign it to $a^{T}$;5:     $y_{j}=\theta c_{j-1}^{s}+\left(1-\theta \right){\tilde{c}}_{s-1}$;6:     $w_{1}=a\left(a^{T}y_{j}-b_{i_{j}}\right)-a\left(a^{T}{\tilde{c}}_{s-1}-b_{i_{j}}\right)+\mu_{s}$;7:     $w_{2}=c_{j-1}^{s}-\eta \times w_{1}$;      // temp variable $w_{1},w_{2}$8:     $c_{j}^{s}=\mathrm{sign}\left(w_{2}\right)\max\{|w_{2}\left|-\frac{1}{\gamma }\eta ,0\right\}$;9:   **end for**10:   ${\tilde{c}}_{s}=\frac{\theta }{m}\sum_{j=1}^{m}c_{j}^{s}+\left(1-\theta \right){\tilde{c}}_{s-1}$;11:   $c_{0}^{s+1}=c_{m}^{s}$;12:**end for****Output:** ${\tilde{c}}_{s}$.

**Theorem** **2** (Non-strongly convex)**.** *If Assumption 2 holds, then by choosing m=Θ(D), the RASVRG$\;{\;}^{\mathrm{NSC}}$ can achieve the following oracle complexity in terms of expectation:*

(17) $$O\left(D\;\sqrt{F\left(c_{0}\right)\;-\;F\left(c^{*}\right)]/\epsilon }+\sqrt{DL{∥c_{0}\;-\;c^{*}∥}^{2}/\epsilon }\right).$$

The proof of Theorem 2 is similar to our previous work [23], and thus it is omitted here. This result indicates that the RASVRG$\;{\;}^{\mathrm{NSC}}$ attained the optimal convergence rate of $O\left(1/S^{2}\right)$, where each epoch included m+D stochastic iterations. We also applied Algorithm 4 to solve the SSC problem. The sparse subspace clustering procedure of the RASVRG$\;{\;}^{\mathrm{NSC}}$ is shown in Algorithm 5.

**Figure 1:**
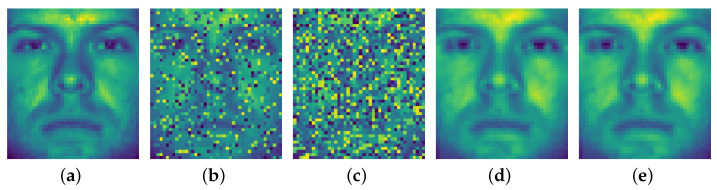
Examples of recovered results of our RASVRG method for a face image with random pixel corruption, where the face image was chosen from the extended Yale B database. (**a**) An original clean image chosen from the extended Yale B database [44]. (**b**,**c**) The images with random pixel corruption of ρ=0.3 and ρ=0.6, respectively. (**d**,**e**) The images recovered by the RASVRG from (**b**) and (**c**), respectively.

We will test whether the RASVRG$\;{\;}^{\mathrm{NSC}}$ works well under the general experimental framework as the algorithm in [22] did. Moreover, we have an intuitive demonstration of the optimization ability of the RASVRG$\;{\;}^{\mathrm{NSC}}$ for computing $c_{j}^{*}$ by recovering the corrupted image. We simply computed the matrix multiplication of $X_{j}$ and $c_{j}^{*}$ to find the restoration of the corrupted image, as shown in Figure 1. All the results show that our RASVRG$\;{\;}^{\mathrm{NSC}}$ performed well for restoration of the corrupted image.
**Algorithm 5** Sparse subspace clustering by RASVRG$\;{\;}^{\mathrm{NSC}}$.**Input:** Date $\mathrm{Data}X=\left[x_{1},\cdots ,x_{N}\right]$ and parameters $k_{\max}$ and ϵ.1:$b\leftarrow x_{j}$, $A\leftarrow X_{j}$, and compute $c^{*}\left(b,A\right)$ with RASVRG$\;{\;}^{\mathrm{NSC}}$;2:Set $C^{*}=\left[c_{1}^{*},\cdots ,c_{N}^{*}\right]$ and $W=\left|C^{*}\right|+\left|C^{*⊤}\right|$;3:Compute segmentation from *W* by spectral clustering;**Output:** Segmentation of data *X*.

For example, we chose an image from the dataset and manually added random pixel corruption to the image with corruption rates ρ = 0.3 and 0.6, as shown in Figure 1b,c. Then, the image was resized to the vector *b*, and the whole dataset without the chosen image was *A*. By using the RASVRG$\;{\;}^{\mathrm{NSC}}$, the recovery vector was then computed as the matrix multiplication of *A* and $c^{*}$. Eventually, the recovery image was resized to a recovered vector. As we can see in Figure 1d,e, our algorithm could recover the damaged image excellently, which shows that the RASVRG$\;{\;}^{\mathrm{NSC}}$ enjoys good robustness intuitively.

## 4. Experimental Results

In this section, we evaluate the efficiency, clustering accuracy and robustness of the RASVRG on many synthetic and real-world face datasets.

### 4.1. Experimental Set-Up

**Datasets.** Firstly, the synthetic datasets were generated as follows. Each element in the matrix $X\;\in \;R^{D\times N}$ was independent and had identical Gaussian distributions. When solving the high underdetermination problem, we found that the performance of all the algorithms was not good, and there was no convergence. For this reason, we generated highly overdetermined data *X* for sparse subspace clustering. For more details, refer to [14]. Test sample *b* could be obtained by $b\;=\;Xc_{0}$, where the sparsity of $c_{0}$ was set to *p*, which means the number of nonzero entries in $c_{0}$ was $p\;\times \;∥c_{0}{∥}_{0}$. Each entry of $c_{0}$ was generated in the interval [−10, 10] according to a uniform distribution. Under the conditions p=0.1 and λ=1e−6, the sparsity of $c_{0}$ was set to p=0.1, which was for consistency with the other literature [14] to compare the results. We computed the relative error of $c_{0}$ as a function of time and the number of passes.

Secondly, the AR face database included more than 4000 frontal images 165×120 in size under different illumination changes, expressions and facial disguises. In order to save computational time, we downsampled the face images and reduced the number of individuals in the experiments. We randomly chose a subset with no more than 15 individuals, and each individual had 26 face images, which were downsampled to 32×32 pixels.

Thirdly, the extended Yale B database contains 2414 images [44]. The face images of each individual were taken under different illumination conditions and cropped to images [45] 192×168 in size. We randomly chose 10 persons, and each individual had 64 images. Each image was manually downsampled to a size of 42×48.

On these three datasets, we compared the clustering accuracy and running time of our algorithms with those of state-of-the-art algorithms under different conditions. For the AR face database, we manually added different levels of random, unrelated block occlusion to measure the performance in the framework of the elastic net. And for the extended Yale B database, different levels of random pixel corruption were added to the images, and the performance was measured in the framework of the $\ell_{0}$/$\ell_{1}$-norm regularizer.

All of the experiments were performed on a computer with a CPU Intel i7-7700K, Windows 7 operating system and 40 GB of memory. We used Matlab and C++ to implement each of our clustering tasks.

**Parameter Settings.** In face clustering tasks, the regularization parameter γ of each algorithm is selected in the range of $10^{\left\{1,2,\ldots ,9\right\}}$. Through tuning γ, all of the algorithms achieve their best performance. The addition of different levels of random pixel corruption and random square blocks is explained in detail. For random pixel corruption, a variable ρ∈[0,1) is introduced as the random corruption index to corrupt randomly chosen pixels from each image, which follows a uniform distribution between [0,1]. Moreover, the occlusion index ϕ∈[0,1] is set. We replaced random square blocks with the unrelated image at a percentage of ϕ. In two real-world face data experiments, through setting each of parameters ρ and ϕ∈[0,1] to 0.3 and 0.6, respectively, we simulated two levels of possible corruption on the clean original image. The occlusion index was set to 0.3 and 0.6 for analysis, and it could also be set to other values, such as 0.1 and 0.8. The smaller the value, the better the recovery result.

### 4.2. Clustering on Synthetic Data

We tested the performance of the RASVRG in the frameworks of the $\ell_{1}$/$\ell_{0}$-norm regularizer and elastic net regularizer in terms of clustering accuracy and running time on synthetic data. All the results reported are the averages of 10 independent trials.

**Figure 2:**
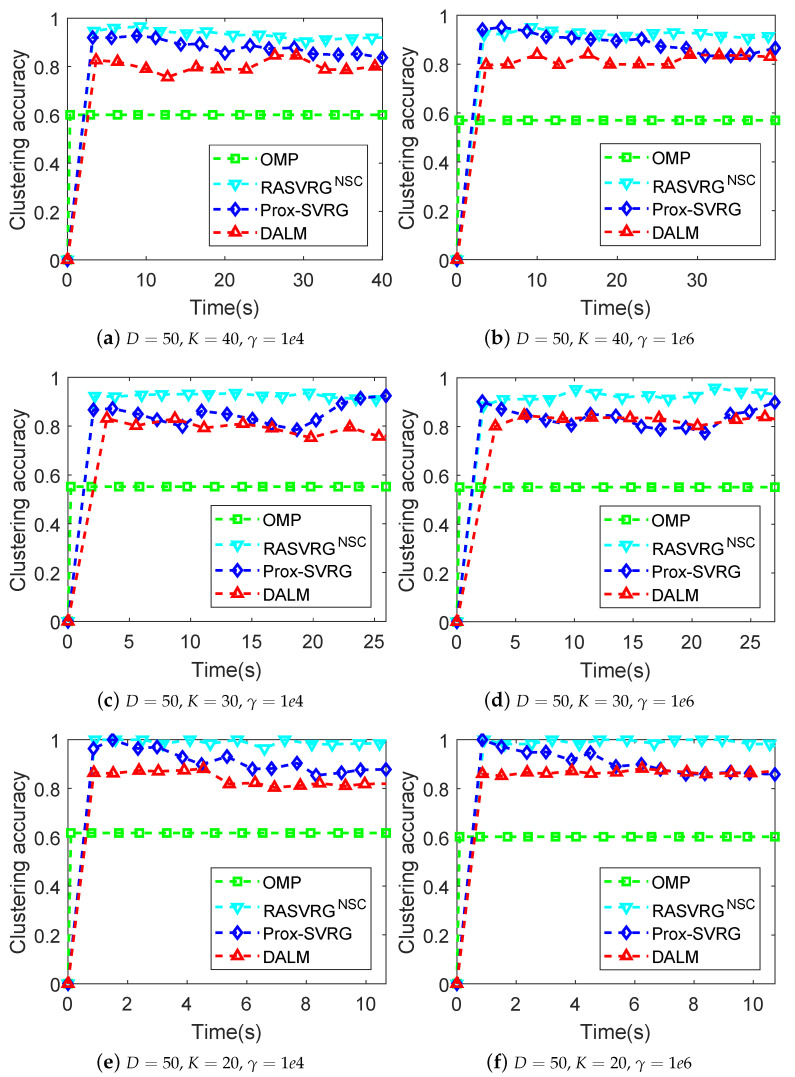
Comparison of the algorithms based on $\ell_{0}$- and $\ell_{1}$-norm regularizers on synthetic datasets under different conditions.

We compared the proposed RASVRG$\;{\;}^{\mathrm{NSC}}$ with three state-of-the-art algorithms namely OMP [22], Prox-SVRG [25] and DALM [46], with $\ell_{1}$/$\ell_{0}$-norm regularizers. The experimental results for the clustering accuracy are shown in Figure 2. Here, *K* denotes the number of subspaces, *D* is the ambient dimension, $N_{i}$ is the number of points per subspace, and *d* denotes the dimension of the subspaces. The clustering accuracies are reported with different *D*, *K* and γ values when setting d=10 and $N_{i}\;\equiv \;60$.

**Figure 3:**
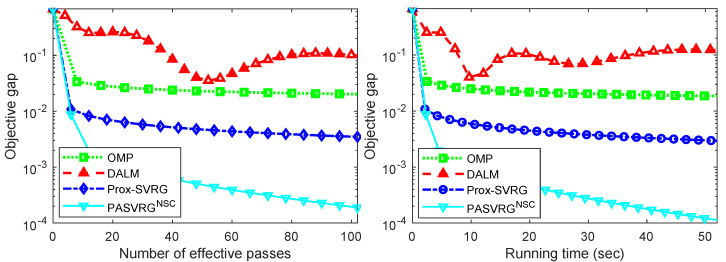
Comparison of the convergence performance of the methods on the synthetic data 50,000 × 1000 in size. Note that the horizontal axis denotes the number of effective passes (**left**) or running time (**right**, in seconds), and the vertical axis corresponds to the objective value minus the minimum value.

From Figure 2, we can see that the RASVRG$\;{\;}^{\mathrm{NSC}}$ consistently achieved accuracy rates over 95% and outperformed other methods, including the Prox-SVRG algorithm. Compared with the Prox-SVRG algorithm, the RASVRG$\;{\;}^{\mathrm{NSC}}$ could achieve a higher accuracy in a shorter time, which also shows the obvious acceleration effect of our RASVRG method. We all know that OMP is a fast algorithm, but its accuracy is usually the lowest. Other algorithms such as the Prox-SVRG algorithm run quickly but ultimately do not reach the highest accuracy. It can be seen that the clustering accuracies of all the algorithms except OMP fluctuated up and down over time. In addition, the accuracies of the Prox-SVRG decreased even after 4 s in Figure 2e, while the RASVRG$\;{\;}^{\mathrm{NSC}}$ had the most stable performance and always achieved superior accuracy over other algorithms. Moreover, it was found that the performance of the DALM and Prox-SVRG algorithms was affected by the change in γ, while the RASVRG$\;{\;}^{\mathrm{NSC}}$ had stable performance. Moreover, we compared the convergence performance (including the objective value minus the minimum value (i.e., objective gap) versus the number of effective passes or running time) of all the methods, as shown in Figure 3. Note that evaluating *N* component function gradients or computing a single full gradient was considered one effective pass. All the experimental results show that the proposed RASVRG$\;{\;}^{\mathrm{NSC}}$ algorithm converged significantly faster than other methods.

We compared the proposed RASVRG$\;{\;}^{\mathrm{SC}}$ with the four algorithms, namely RFSS [47], FISTA [48], Homotopy [49], and the Prox-SVRG [25], for the elastic net model. In order to evaluate the robustness of different methods versus the parameter γ, we varied γ in the range of $10^{\left\{5,6,7,8,9\right\}}$ when setting d=40, $N_{i}\;\equiv \;60$ to generate two types of random data.

The clustering accuracies of these methods are listed in Table 1. It is clear that the RASVRG$\;{\;}^{\mathrm{SC}}$ achieved the highest accuracy in most cases. The stochastic method (Prox-SVRG) performed relatively well, but the running time was longer than that of the RASVRG$\;{\;}^{\mathrm{SC}}$. The accuracies of RFSS and Homotopy were similar and much lower than those of the RASVRG$\;{\;}^{\mathrm{SC}}$ and Prox-SVRG, and thus we did not report their running time results. FISTA performed much better than Homotopy and RFSS, and it sometimes achieved an accuracy of over 90%, but it ran more than five times slower than the RASVRG$\;{\;}^{\mathrm{SC}}$. All the results indicate that the RASVRG$\;{\;}^{\mathrm{SC}}$ was superior to the other methods in terms of both robustness and efficiency.

### 4.3. Face Clustering Based on Elastic-Net Regularizer

In this part, we compare the proposed RASVRG$\;{\;}^{\mathrm{SC}}$ with popular elastic net solvers on the AR face database, including regularized feature sign search (RFSS) [47], Homotopy [49], the proximal stochastic variance reduction gradient (Prox-SVRG) [25] and the fast iterative shrinkage thresholding algorithm (FISTA) [48]. In each trial, we randomly picked k∈{2,5,8,11,14} individuals and took all the images (under different illuminations) as the data to be clustered.

Table 2 reports the clustering accuracies of different methods on the face datasets with manually added random, unrelated block occlusion. All the results indicate that in most cases, the RASVRG$\;{\;}^{\mathrm{SC}}$ outperformed the other algorithms in terms of clustering accuracy. When the number of clusters was k=2, all the algorithms except for Homotopy and RFSS achieved relatively high accuracies. With the number of clusters and the occlusion rate ϕ increasing, the performance of Homotopy had certain advantages over other methods, except for the RASVRG$\;{\;}^{\mathrm{SC}}$. In addition, the accuracies of Homotopy were close to those of RFSS. Due to the existence of random block occlusion, the clustering accuracies of all of the algorithms decreased rapidly as the number of clusters *k* increased. When k≤10, the RASVRG$\;{\;}^{\mathrm{SC}}$ obtained the best clustering accuracy. In the case of slight corruption (e.g., ϕ=0.3), the Prox-SVRG algorithm achieved the best accuracy when k=14, but in the other cases, the RASVRG$\;{\;}^{\mathrm{SC}}$ significantly outperformed the other algorithms. When the occlusion rate was high (e.g., ϕ=0.6), the RASVRG$\;{\;}^{\mathrm{SC}}$ performed much better than the other algorithms, except for the case of k=14.

### 4.4. Face Clustering Based on $\ell_{0}$- and $\ell_{1}$-Norm Regularizers

In this part, we evaluate the clustering accuracy and robustness of the RASVRG$\;{\;}^{\mathrm{NSC}}$ on the extended Yale Face B database compared with five popular $\ell_{0}$/$\ell_{1}$-based solvers, namely the Prox-SVRG [25], Homotopy [50], OMP [22], DALM [46] and PALM [46]. We randomly picked k∈{2,3,5,8,10} individuals and took all the images under different illuminations as the data to be clustered.

**Figure 4:**
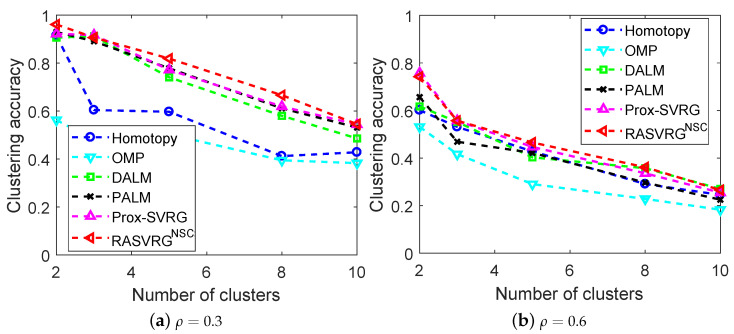
Comparison of the algorithms based on $\ell_{0}$- and $\ell_{1}$-norm regularizers on the extended Yale B database with different random pixel corruption.

The clustering performance of all the methods is shown in Figure 4. The RASVRG$\;{\;}^{\mathrm{NSC}}$ attained the highest clustering accuracy in almost all cases. It is quite clear that the performance of OMP was the worst in all cases when the random pixel corruption value ρ varied from 0.3 to 0.6. In the case of slight pixel corruption (e.g., ρ=0.3), all the algorithms reached more than 90% accuracy when k=2, except for OMP. The accuracy of Homotopy decreased rapidly when k>2. The Prox-SVRG and PALM had rather close accuracies and maintained high clustering accuracies as *k* increased. When the random pixel corruption (e.g., ρ=0.6) was high, only the RASVRG$\;{\;}^{\mathrm{NSC}}$ and Prox-SVRG algorithms achieved more than 70% accuracy when k=2. As the number of clusters increased, the clustering accuracies of all of the algorithms decreased. DALM performed the best when k=10. Similarly, the RASVRG$\;{\;}^{\mathrm{NSC}}$ performed well under various numbers of clusters, and the clustering accuracy was always about 10% higher than that of Homotopy.

The RASVRG algorithm outperformed other algorithms in terms of clustering accuracy under the frameworks of both elastic net and $\ell_{1}$/$\ell_{0}$-norm regularizers on the real-world face datasets with manually added random pixel corruption and unrelated block occlusion in most cases. This illustrates the strong robustness and wide applicability of our algorithms for various SSC problems, especially robust face clustering.

## 5. Conclusions and Future Work

In this paper, we proposed two efficient algorithms, the RASVRG$\;{\;}^{\mathrm{SC}}$ and RASVRG$\;{\;}^{\mathrm{NSC}}$, to solve the elastic net regularizer and $\ell_{1}$-norm regularizer-based sparse subspace clustering problems, respectively. To the best of our knowledge, this work is the first one to propose faster stochastic optimization algorithms instead of deterministic methods to solve various large-scale SSC problems, especially large-scale robust face clustering. The experimental results for both synthetic and real-world face datasets demonstrated the effectiveness of our algorithms. Our algorithms performed much better than the state-of-the-art methods in terms of both clustering accuracy and running time. On the synthetic datasets, both the RASVRG$\;{\;}^{\mathrm{NSC}}$ and RASVRG$\;{\;}^{\mathrm{SC}}$ achieved more stable and higher clustering accuracies in most cases compared with other elastic net solvers and $\ell_{1}$-norm solvers. On the real-world face datasets with different levels of random pixel corruption and random block occlusion, our algorithms also achieved much higher clustering accuracies, which indicates their robustness to corrupted or damaged data. In other words, aisde from enjoying a higher speed, the RASVRG algorithm performed much better than the state-of-the-art methods in terms of both accuracy and robustness.

It was noticed that the RASVRG tracked only one variable in the inner loop, which made it quite friendly to asynchronous parallel and distributed implementation, including privacy-preserving federated learning [51]. Applying parallel acceleration to our algorithms and other subspace clustering problems, such as those in [52,53], can make the RASVRG excellent in terms of both clustering accuracy and speed in large-scale privacy-preserving clustering. This can be an exciting orientation for our future work.

## Figures and Tables

**Table 1:** Clustering accuracy and running time of the algorithms based on elastic net regularizers on synthetic data. The highest clustering accuracy is shown in bold.

γ	$10^{9}$	$10^{8}$	$10^{7}$	$10^{6}$	$10^{5}$
(**a**) D=50, K=20					
	Clustering accuracy (%)				
RFSS	73.38	81.58	78.40	70.88	71.10
FISTA	90.65	92.65	88.96	91.75	89.03
Homotopy	76.18	79.65	74.90	73.21	71.56
Prox-SVRG	94.81	98.21	96.18	97.30	95.88
RASVRG$\;{\;}^{\mathrm{SC}}$	**97.33**	**98.63**	**98.76**	**98.81**	**96.06**
	Running time (seconds)				
FISTA	7.61	10.46	10.15	10.63	9.82
Prox-SVRG	1.86	1.74	1.77	2.03	2.05
RASVRG$\;{\;}^{\mathrm{SC}}$	**1.31**	**1.34**	**1.32**	**1.46**	**1.49**
(**b**) D=50, K=40					
	Clustering accuracy (%)				
RFSS	69.78	72.78	71.31	71.82	70.50
FISTA	87.01	87.85	88.95	86.51	87.50
Homotopy	74.41	75.70	76.33	71.43	71.01
Prox-SVRG	92.85	91.60	93.28	92.89	93.64
RASVRG${\;}^{\mathrm{SC}}$	**94.25**	**94.85**	**93.32**	**94.17**	**94.79**
	Running time (seconds)				
FISTA	11.86	18.75	22.63	23.11	20.24
Prox-SVRG	3.60	3.88	4.70	4.21	4.36
RASVRG$\;{\;}^{\mathrm{SC}}$	**2.77**	**3.02**	**3.23**	**3.16**	**3.26**

**Table 2:** Clustering performance of different algorithms based on the elastic net regularizer on the AR face database with random, unrelated block occlusion.

Clusters (*k*)	2	5	8	11	14
(**a**) ϕ=0.3					
	Clustering accuracy (%)				
RFSS	53.84	33.84	25.00	18.88	17.58
FISTA	55.76	33.07	24.51	19.93	17.85
Homotopy	53.84	33.84	24.03	18.88	17.58
Prox-SVRG	61.53	32.30	24.51	20.97	19.27
RASVRG$\;{\;}^{\mathrm{SC}}$	**65.38**	**35.14**	**25.31**	**21.67**	**19.38**
(**b**) ϕ=0.6					
	Clustering accuracy (%)				
RFSS	53.84	30.00	22.11	19.58	18.40
FISTA	55.76	28.46	21.15	18.53	15.93
Homotopy	53.84	29.23	23.07	20.62	19.23
Prox-SVRG	57.69	30.00	22.11	19.93	16.75
RASVRG$\;{\;}^{\mathrm{SC}}$	**57.69**	**31.14**	**23.41**	**21.52**	**20.94**

## Data Availability

Data are contained within the article.
